# Prostatic Enlargement with Some of the Causes and Complications

**Published:** 1907-06

**Authors:** W. R. Kone

**Affiliations:** Oklahoma City, Okla.


### TEXAS MEDICAL JOURNAL.

Established July, 1885.

F. E. DANIEL, M. D., - - - - - Editor, Publisher and Proprietor.

PUBLISHED MONTHLY.—SUBSCRIPTION $1.00 A YEAR.

VOL. XXII. AUSTIN, JUNE, 1907. No. 12.

The publisher is not responsible for the views of contributors.

For Texas Medical Journal.

### Prostatic Enlargement With Some of the Causes and Complications.

BY DR. W. R. KONE, OKLAHOMA CITY, OKLA.

In selecting this subject for our consideration, I have not done so thoughtlessly, nor neither have I done so disregarding the many manifold puzzling problems which may confront us. For, as we read the different literature on the subject, and think of gonorrhea as a causative agent in producing prostatitis, and many other things which may enter as a cause or sequel, our minds almost seem to go out into space. But when we begin to particularize this and that from a pathological or clinical standpoint our subject assumes a more tangible form and is on a better working basis.

While I believe a great majority of those cases come from a gonorrheal origin, yet observation forces us to the conclusion that there are other factors that go to make up a clinical case and which should receive due consideration. Be this in the future as in the past, we have no moral right to sneer at the unfortunate victim who, through the laws of consequence, victimizes himself to fall a prey to disease; for, if only those who were not in any way tainted with the subject under discussion were admitted to respectable society, a large majority of the human race would be ostracized. This being the case, we are brought face to face with the fact of our responsibility of treating those cases to the best of our ability, it makes no difference from what source the disease is contracted.

Over-indulgence in sexual intercourse may be considered as a potential factor in producing prostatic enlargement in the young man as well as in the old; but it is in the older patients that we expect most frequently to meet these cases; as in the old man who marries a young girl in her teens, and through the covenant of his situation renews his vigor with an energy greater than he can stand, and is regarded by the laity as victimizing himself to fall a prey to what he surveys, and in the end reaps the reward of over-indulgence. While I think this does happen to the old man from his increased sexual desire, and in a sense is true to nature, and is logically correct, yet is it not just as logical to suppose that hypertrophied prostates, or a fibrous condition sometimes found in these older people, would stand as an underlying cause and would be capable of increasing this abnormal sexual desire, as to blame the old man for it all? We had an inkling of this truth (if it be one) brought out in a paper read before the Southwestern Medical Society which recently convened here in this city, when it was said that in a small minority of the cases operated on the prostates were in a shrunken, fibrous condition, and were not prostatic enlargements.

Then, again, other prevailing factors which we are not as well acquainted with may, at last, be the main reason, and will in a large measure account for this functional activity of the generative organs in these senile cases, and that is: The capsule that surrounds the prostates becomes fibrous and contracted as age advances and causes a reflex sensitive condition of the prostatic portion of the urethra; or, a titillating of the nerves that go to supply the pendicular portion of the penis, and in a way causes erection to take place that through opportunity would lead on to the goal. Whether this be the cause or some other proverbial mystery be hidden away in the researches of time, I know not; but there is one thing I think it would be safe to say that, whatever conclusion we may come to, the disease is preceded by inflammation of some kind.

In a case of prostatic enlargement sufficient to produce any degree of urethral obstruction, we would have as a natural consequence complications that would likely give the patient as much or more trouble than the disease itself; and we would have to consult those sequels in treating the disease. Should we be asked to name the complications that we would most likely have to encounter in treating a case of prostatic enlargement, I would mention cystitis, retention of the urine (in all its varieties), calculus, Bright's disease and uremia. To avoid cystitis should be one of our first efforts in treating a case. It comes on so quickly after instrumental manipulation that we have learned to regard catheter infection as the most prolific source of cystitis; and it is a sad fact for which we have no remuneration, coming as it does as a feature of enlarged prostates, the surgeon spends more time and greater anxiety in relieving the cystitis than anything else until he gets it under his control.

Retention of urine, whether complete or partial, is capable in itself in itself of exciting cystitis, and it is in these cases with enlarged prostates, where we have partial obstruction, that we would expect to first find residual urine. The urine undergoes some chemical changes, either ammoniacal, or excess of salts or lime, which in time will cause a cystitis. A close observer may gain many points that will be decidedly to his advantage in forming a correct conclusion as to the character of the urine as well as the quantity that is in the bladder. A careful analysis should always be made in these cases to find the true condition of the kidneys. While no one at the present time would undertake to deny the value of the microscope of its usefulness, yet I would far prefer knowing the amount of urine passed in the preceding twenty-four hours, with the amount of urea in a given time, than to know all about the tubes, casts, or even albumen. In a case of complete retention of urine, we should not be surprised to find uremic symptoms, or that the kidneys themselves have become seriously and probably permanently crippled by the damming-back process through the ureters to the kidneys. Catheterization is the means most usually thought of when the bladder is distended, with inability to empty itself. This usually is an easy matter, provided there is no false passage made from rough manipulation, or where the prostates are not too large.

There are those cases where we have an incomplete retention of urine, the residual urine being considerable, and yet every hour or so the patient may pass a fair quantity of urine. This is the overflow of an abnormally distended bladder, and where it reaches a certain amount with increased pressure causes a relaxation of the sphincter muscle until the pressure is lowered.

There is another class of cases following enlarged prostates, or, without the prostates being involved at all, where the bladder becomes so irritable as to hold only a small amount of urine, compelling the patient to void his urine every few minutes. These cases are entirely different from the preceding, and no mistake should be made. The symptoms closely simulate each other in some particulars.

The inability to pass a catheter on account of a tight stricture often sets our plans at sea and leaves us to grapple for other appropriate means, which in turn will give relief. Rectal examination should at once be resorted to in these cases to determine if the prostates are enlarged and, if so, to what degree. The seminal vesicles should also be palpated while the fingers are engaged in the rectum and a note made as to whether or not any hemorrhoids are present. It often takes a long finger to gain this information in a satisfactory way, and if the bladder is empty, and no cystitis present, by gently laying the hand over the bladder and pressing downward, additional advantage is gained. If retention of urine in any degree has been found for any length of time, veins, engorgements of the prostates, the vessels of the neck of the bladder, and prostatic plexus, any or all, may be involved, which only goes to show a more serious case, to say nothing about the real condition of the bladder itself. Who could stay the right of his opinion to the patient, when he lies there harassed by pain, which almost amounts to torture, calling for something to be done almost every minute. Have we no guide to look to, that will stand between us and all grave mistakes that we are likely to make, and in this way hold out hope and encouragement to satisfy the hungry mind of our sorely afflicted patient who fears that death is too near the door to hope for better days.

I would not care to deny or confirm the last question without hinging my decision on a correct understanding and to know the relation the prostate sustains towards the bladder in an abnormal way; and whether or not the mucous membrane of the urethra passing through the prostatic portion has its findings other than in a healthy condition. Should circumstances shroud us with the conviction, based on other than what we know to be a fact, I would be inclined to shrink away from the main question and in the end answer in the negative. But we know that optimistic views in practicing medicine go a long way in some cases, and those cases where the disease of the mind is as much or more at fault than the parts themselves furnishes the best medium of our assurance of success. Then, again, on examination where we find little or no prostatic trouble, and that the urethra in this portion is not damaged to any notable degree, and as a whole amenable to treatment, and where the whole trouble seems to hinge itself on bladder infection; if encouragement is what we want, a positive assurance can be given our patient for a speedy recovery. But, mark you, our path in treating these diseases, as well as in making a correct diagnosis, is not always so easy, as the picture cited above, nor neither is our undertaking so flattering at times as we would have it, for instance, an enlarged, boggy prostate where it is pressing up against the bladder, that is sufficient to cause a ball valve shape to act as a cut-off in part or altogether the urine from the urethra, and there forming post pockets behind the prostates for retention and decomposition of urine. I say what could we expect for our patients other than some operative relief? It is not all so good and so true that we do not have to spare patience and time to claim the victory in the race; and yet, on the other hand, he who dallies with these diseases without in some degree understanding them will be found, in the end, doubting. It is not the mysteries of the future that should concern us the most, but rather the conditions of the present, while opportunity is afforded.

So far in this paper I have dealt very lightly on gonorrhea as being one of the factors in producing prostatic troubles. I have said that the majority of our cases comes from this cause without stating my real belief in the matter. My convictions are hinged principally on the old or neglected cases of gonorrhea, where gleet has asserted itself, and the patient is unable from some cause or other to rid himself of this disease. It may be that it has found its lodging place just behind a stricture where the urethra has become enlarged from pressure, affording, as it does, a secluded spot as a hiding place, and days may come and go and it will stand as it has stood through the many trials to break up the nesting place of the hidden monster and from whence it refuses to be driven from its hunting ground, and no more be allowed the freedom of action to scatter the mighty poison to parts further on.

Or, again, it may take up its abode in the little ducts that we find here and there in the prostatic portion of the urethra, as the ejaculatory duct passing back into the seminal vesicles or vas deferens on its way to the testes to harass the patient here for days to come. Or has it not been seen in the mouths of the sinus poculosis, where it has waged its battle and not been found wanting, as evidenced by little red lines and swollen mouths on each side of the verumontanum?

It is the nature of the little intruder to set up monuments all along the line of his work, so if any unfriendly hand seeks to find new fields undiscovered, he will sit down and hide his face with shame with a wish that he had never been born as a pioneer to hunt out new fields undiscovered on the plains of the prostates. There is no place in the range of possibility where it does not go, from the most fascinating lady in the land who is suffering with gonorrheal pyo-salphinx to the old man who is tottering towards the grave suffering from prostatitis or gonorrheal rheumatism.

Listen to the prediction of the prodigality of virtue as it wanders off into vice, stated by such men as Morrow, Mann, Lewis and Ponery. H. S. Pomeroy, of Boston, declares in the last thousand years civilized society has been cursed by four great physical evils: smallpox, the so-called white plague, alcoholism and the social evil. The ingenious Dr. Jenner has put smallpox in the back ages; the hand of repentance has been laid on the white plague and the alcoholic evil; but, on the fourth, the social evil, we need more light.

Dr. Bransford Lewis, of St. Louis, when speaking of the race as a whole, says that 85 per cent of the gonorrhea in the married women is contracted innocently from their husbands; he also holds that 80 per cent of blindness is due to the same cause; and that 90 per cent of the men have some time in their life had gonorrhea. Mann holds that about 70 per cent of all abdominal pelvic operations which the surgeon is called upon to treat are due to gonorrhea, and almost in the same breath says that were it possible to eliminate gonorrhea from the human race the gynecologist would sink almost into insignificance.

Is this not an amazing statement to be read before a medical society, and is it not a fact that he who reads between the lines and marks the onward march of gonorrhea is keeping pace with the modern times more nearly than the man that affirms that venereal diseases are about on a standstill. Life is not today as it was yesterday; he that diddles in dastardly places has no guarantee on his organs remaining sound and whole, whether it involves the prostates primarily or comes on in after years.

